# *N*-Acyl Homoserine Lactone-Mediated Quorum Sensing in *Aeromonas veronii* biovar sobria Strain 159: Identification of LuxRI Homologs

**DOI:** 10.3389/fcimb.2016.00007

**Published:** 2016-02-16

**Authors:** Xin-Yue Chan, Kah-Yan How, Wai-Fong Yin, Kok-Gan Chan

**Affiliations:** Division of Genetics and Molecular Biology, Faculty of Science, Institute of Biological Sciences, University of MalayaKuala Lumpur, Malaysia

**Keywords:** *Aeromonas veronii*, quorum sensing, autoinducer, *N*-butanoylacyl homoserine lactone, liquid chromatography mass spectrometry (LC-MS)

## Background

Quorum sensing (QS) is the term which refers to bacterial cell-cell communication to coordinate a diverse array of physiological behaviors in the entire communities. This signaling system allows the bacteria to sense its population density in response to the concentration of signaling molecules (Miller and Bassler, 2001). Unlike Gram-positive bacteria which employ oligopeptide/two component-type QS circuit, Gram-negative bacteria use the diffusible *N*-acylhomoserine lactones (AHLs) signaling molecules to mediate expression of many phenotypes and cellular activities (Federle and Bassler, 2003).

AHLs are widely conserved signal molecules; they consist of the same homoserine lactone moiety but differ in the length and structure of acyl side chain at the C3 carbon (Fuqua and Greenberg, 2002). With such structural molecules, AHLs are generally amphipathic in nature. They are water-soluble and able to diffuse freely across the phospholipid cell membranes (Pearson et al., 1999). AHL-dependent QS system comprises three principal components: (i) the AHL signaling molecules, (ii) AHL synthase protein, LuxI to produce AHLs, and (iii) a transcriptional regulator protein, LuxR to bind to AHLs (Shrout and Nerenberg, 2012). Accumulation of AHLs above a threshold concentration triggers the formation of signal-receptor protein complexes which in turn activate the expression of appropriate target genes (McClean et al., 1997; Eberl, 1999). In the past decade, numerous studies have shown the importance of AHLs in regulation of a range of biological functions such as biofilm formation, antibiotic production, swarming motility, conjugation, and sporulation. The basic mechanisms of AHL synthesis and regulation are found to be conserved in many species of proteobacteria despite expression of different target phenotypes (Miller and Bassler, 2001; Dong et al., 2002). In addition, each QS-regulated target gene requires a specific cell density in order to be activated and there is no such single population density which could trigger expression of all genes (Schuster et al., 2003).

*Aeromonas* spp. are Gram-negative, facultative anaerobic, rod-shaped bacteria belonging to the family Aeromonadaceae. They are ubiquitous bacteria which thrive in many terrestrial and aquatic environment (Daskalov, 2006). This genus consists of psychrophiles and mesophiles which are both primary and opportunistic pathogens of cold and warm-blooded animals. Recently, *Aeromonas* spp. have gained more clinical recognitions as they are commonly associated with food and waterborne diseases (Ansari et al., 2011). The most important pathogens are *A. hydrophila, A. caviae*, and *A. veronii* biovar *sobria*. These species are primary causative agents of both gastrointestinal and extra-intestinal infectious diseases (Vila et al., 2002). In fact, *A. veronii* is often associated with traveler diarrhea (Gröbner et al., 2007).

Recently, our group has isolated 22 *Aeromonas* strains from various clinical samples i.e., bile, blood, peritoneal fluid, pus, stool, and urine, and their QS activities were investigated. Among the *Aeromonas* isolates, *A. veronii* biovar sobria strain 159 (hereafter referred to as strain 159) isolated from a stool sample was found to exhibit QS activities (Chan et al., 2011). Whole genome sequencing of this bacterium was performed and it was found that this strain shares a high degree of genome synteny with *A. hydrophila* ATCC 7966. Upon analysis and genome annotation, a pair of LuxIR homologs, termed as AveIR, was found to be located in Contig 47. The AHL profile was then obtained from the culture supernatant of strain 159 using liquid chromatography mass spectrometry (LC-MS).

## Materials and methods

### Bacterial source, isolation and culture

*A. veronii* strain 159 was isolated from the stool of a patient admitted to University of Malaya Medical Center, Malaysia. This bacterium was maintained aerobically in LB (Luria Bertani, Merck) medium at 37°C. Strain 159 was also stored frozen at −70°C in 50% (v/v) glycerol.

### Gene annotation and phylogenetic analysis

Gene annotation was performed using the SEED-based automated annotation system provided by the Rapid Annotations using Subsystems Technology (RAST) server (version 4.0) (Aziz et al., 2008) to look for the presence of LuxIR homologs. The protein sequences of both AveI and AveR were compared with GenBank databases using BLASTX program available from NCBI website (http://www.ncbi.nlm.nih.gov/). Ten LuxI and LuxR homologs with the highest similarities were chosen. Redundant sequences or bacteria strains with ambiguities were omitted. A phylogenetic trees corresponding to both proteins were constructed using Molecular Evolutionary Genetic Analysis (MEGA) version 5.2 (Tamura et al., 2011). Neighbor joining algorithm was used with boostrap value of 1000, expressed as percentage of 1000 replicates.

### Extraction of AHL

*A. veronii* strain 159 was grown in LB medium buffered to pH 6.5 with 50 mM of 3-[*N*-morpholino] propaneusulfonic acid (MOPS) to prevent degradation of AHLs (Yates et al., 2002). The bacterium was grown at 37°C with agitation at 220 rpm. The spent culture supernatant was extracted thrice with equal volume of acidified ethyl acetate (0.1% v/v glacial acetic acid in ethyl acetate, Merck, Germany). The ethyl acetate extracts were evaporated to dryness in fume hood. The dried extracts were then resuspended in 1 mL of acidified ethyl acetate and allowed to dry again. Then, 1 mL of acetonitrile (HPLC grade, Merck, Germany) was added to dissolve the extracted AHLs. The mixture was then filtered and 100 μL of aliquot was withdrawn and placed in sample vials for analysis by liquid chromatography mass spectrometry (LC-MS).

### Identification of AHL molecules by mass spectrometry (MS)

The AHL prrofile of strain 159 was obtained by High Resolution Tandem Triple Quadrupole Mass Spectrometry (LC-MS/MS) System according to previously reported method (Wong et al., 2012). LC delivery system using Agilent 1290 Infinity system (Agilent Technologies Inc., Santa Clara, CA, USA) was employed with Agilent ZORBAX Rapid Resolution HT column (2.1 × 50 mm, 1.8 μm particle size). Both mobile phases A and B were 0.1% v/v formic acid in water and 0.1% v/v formic acid in acetonitrile, respectively. The parameters of the gradient profiles were set as followed (time: mobile phase A: mobile phase B): 0 min: 60:40, 5 min: 20:80, 7 and 10 min: 5:95, and 11 and 13 min: 60:40. Two microliter of sample was injected and the analysis was performed using a flow rate of 0.3 mL/min at 37°C. The Agilent 6490 Triple-Quad LC-MS/MS system was used to perform the high-resolution electrospray ionization mass spectrometry (ESI-MS) in positive mode. The probe capillary voltage was set at 3 kV, sheath gas at 11 mL/h, nebulizer pressure at 20 psi and desolvation temperature at 250°C. Nitrogen gas was used as the collision gas in the collisionally-induced dissociation mode for the MS/MS analysis and the collision energy was set at 10 eV. The Agilent MassHunter software was used to analyse the MS data (Yin et al., 2012; How et al., 2015). Known amounts of synthetic AHLs (Sigma, St. Louis, MO) were injected as standards.

## Results

**Figure 1:**
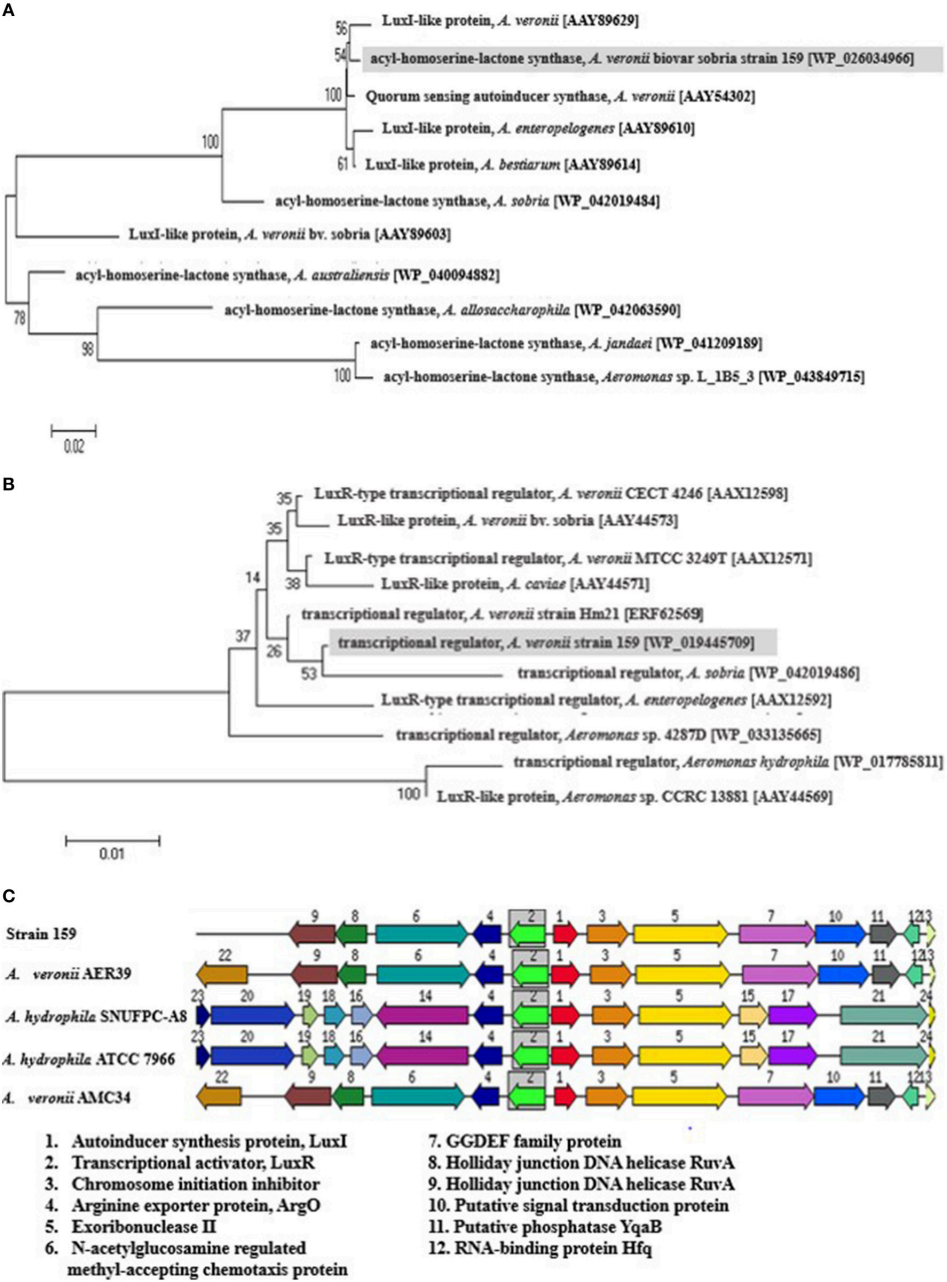
**Phylogenetic tree of LuxIR homologs and the organization of the gene cluster**. Phylogenetic tree describing evolutionary distances between **(A)** LuxI homologs and **(B)** LuxR homologs from different *Aeromonas* species using Neighbor-Joining algorithm. The tree is drawn to scale, with branch lengths in the same units as those of the evolutionary distances used to infer the phylogenetic tree. The horizontal bar at the bottom represents mean number of substitutions per site. The bootstrap values are expressed as percentages of 1000 replications. **(C)** The organization of LuxIR homologs in strain 159 and closely related species. The gene clusters of LuxI/R homologs in strain 159 were compared with closely-related species, *A. hydrophila* subsp. *hydrophila* ATCC 7966, *A. veronii* AMC34, *A. veronii* AER39, and *A. hydrophila* SNUFPC-A8. Homologous proteins are shown with the same color and arrows indicate the relative orientations of the genes. GenBank accession numbers (in parentheses): LuxI-like protein, *A. veronii* [AAY89629]; quorum sensing autoinducer synthase, *A. veronii* [AAY54302]; LuxI-like protein, *A. enteropelogenes* [AAY89610]; LuxI-like protein, *A. bestiarum* [AAY89614]; acyl-homoserine-lactone synthase, *A. sobria* [WP_042019484]; LuxI-like protein, *A. veronii* bv. sobria [AAY89603]; acyl-homoserine-lactone synthase, *A. australiensis* [WP_040094882]; acyl-homoserine-lactone synthase, *A. allosaccharophila* [WP_042063590]; acyl-homoserine-lactone synthase, *A. jandaei* [WP_041209189]; acyl-homoserine-lactone synthase, *Aeromonas* sp. L_1B5_3 [WP_043849715]; LuxR-type transcriptional regulator, *A. veronii* CECT 4246 [AAX12598]; LuxR-like protein, *A. veronii* bv. sobria [AAY44573]; LuxR-type transcriptional regulator, *A. veronii* MTCC 3249T [AAX12571]; LuxR-like protein, *A. caviae* [AAY44571]; transcriptional regulator, *A. veronii* strain Hm21 [ERF62569]; transcriptional regulator, *A. sobria* [WP_042019486]; LuxR-type transcriptional regulator, *A. enteropelogenes* [AAX12592]; transcriptional regulator, *Aeromonas* sp. 4287D [WP_033135665]; transcriptional regulator, *A. hydrophila* [WP_017785811]; LuxR-like protein, *Aeromonas* sp. CCRC 13881 [AAY44569].

Whole-genome shotgun sequencing of *A. veronii* strain 159 was performed recently using Illumina HiSeq 2000 (Illumina, Inc., CA) platform (Chan et al., 2012). The genome sequence has been deposited at DDBJ/EMBL/GenBank under the accession no. ALOT00000000. From RAST analysis of the draft genome, a copy of each LuxI and LuxR homologs were found, termed as AveI and AveR, respectively. Both protein sequences were deposited in NCBI with GenBank accession number WP_026034966.1 and WP_019445709.1. The phylogenetic tree illustrated that both AveI and AveR proteins share high homology with their reported counterparts from other *Aeromonas* species, indicating that they may share some ancestry relationship and the proteins are conserved throughout evolution (Figures 1A,B). The analysis revealed that LuxI homolog from strain 159 is more closely related to LuxI homologs from *A. veronii* strains [AAY89629 and AAY54302]. It falls within a clade that includes *A. bestiarum* [AAY 89614] as well as *A. enteropelogenes* [AAY 89610]. On the other hand, transcriptional regulator, AveR of strain 159 shows closer affinity to *A. sobria* [WP_042019486] than to *A. veronii* [ERF62569, AAX12571, and AAX12598]. The high degree of homology suggests that the LuxI/R are conserved throughout evolution among *Aeromonas* species and these proteobacteria may possibly share similar basic QS mechanism and gene regulation in AHL production even though their expression target genes could be different.

From *in silico* analysis of strain 159 and closely-related species, all *Aeromonas* spp. are found to possess single copy of both *aveI* and *aveR*, which are 639 bp and 777 bp genes, which encode for 212 and 258 amino acids, respectively. RAST analysis revealed that *aveIR* gene cluster of strain 159 shares high homology with its closest counterpart, *A. hydrophila* ATCC7966. Both autoinducer synthesis proteins and transcriptional activator proteins are found in reversed orientation. In the vicinity of LuxI/R homologs are genes encoding chromosome initiation inhibitor, arginine exporter protein, and exoribonuclease II enzyme. Chromosome initiation inhibitor is a DNA-binding protein that inhibits chromosome replication while exoribonuclease II involves in mRNA degradation. Such organization of gene cluster is also found in *A. hydrophila* and *A. salmonicida* (Swift et al., 1997). It could be possibly that the regulation of the genes responsible for cell divisions are regulated by LuxI/R system in these *Aeromonas* species. In strain 159, *aveI* and its cognate *aveR* partner are adjacent genes that are 62 bp apart, similar to *A. hydrophila* ATCC7966 (Figure 1C). In between, a region of dyad symmetry known as the *lux* box was identified. Surprisingly, the *lux* box was found to be identical to the one from *A. veronii* MTCC 3249 but shorter than *A. hydrophila* and *A. salmonicida* (Jangid et al., 2012).

The amino acid sequence of AveI was analyzed using InterPro (http://www.ebi.ac.uk) to identify conserved protein domain and molecular function. Results from InterPro shows that AveI fulfills the criteria as an AHL synthase as it possesses autoinducer synthesis and acyl-CoA *N*-acyltransferase conserved sites. Meanwhile, InterPro revealed that AveR protein consists of both N-terminal autoinducer binding domain and C-terminal DNA-binding domain of LuxR-like protein which are the fundamental requirements of a functional LuxR transcriptional receptor (Tsai and Winans, 2010).

**Figure 2:**
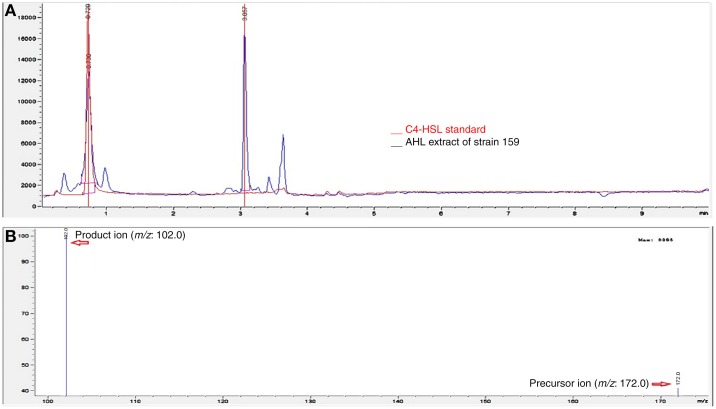
**MS analyses showing the AHL profile of strain 159 using LC-MS/MS**. **(A)** Overlaid of the chromatogram of synthetic standard of C4-HSL with the chromatogram of culture supernatant from strain 159 at retention time of 0.7 min. **(B)** Spectrum extracted from the selected peak of chromatogram for strain 159 at 0.7 min shows the presence of C4-HSL product ion (*m/z*: 102) and precursor ion (*m/z*: 172).

In our previous study (Chan et al., 2011), a bioassay using thin layer chromatography demonstrated that strain 159 secreted C4-HSL into the growth media. Hence, in this study, we used Triple-Quad LC-MS/MS to determine the AHL profile of extracted culture supernatant of strain 159. The chromatogram of strain 159 was overlaid with the chromatogram from the synthetic standard at 0.7 min (Figure 2). The presence of C4-HSL with *m/z* value 172.000 was indistinguishable to the corresponding synthetic compounds at their respective retention times. The product ion of *m/z* 102 corresponds to the presence of lactone ring moiety of C4-HSL. Long chain AHL was not secreted by strain 159. This was in agreement with the recently published data by Chan et al. (2011).

Among *Aeromonas* species, most of the studies on QS were substantially found from *A. hydrophila* (AhyIR) and *A. salmonicida* (AsaIR) (Swift et al., 1997, 1999). Both *Aeromonas* species are well-known pathogens of humans and fish: *A. hydrophila* is the etiological agent for aeromonad septicemia while *A. salmonicida* is responsible for furunculosis in salmonid fish (Fryer and Bartholomew, 1996). Studies have demonstrated that AhyIR plays essential roles in biofilm formation, exoprotease production and type IV secretion system (Swift et al., 1999; Khajanchi et al., 2009) while AsaIR is possibly implicated to regulation in cell division (Swift et al., 1997). Hence, it is of interest to get further insights of the roles played by AveIR in strain 159.

According to a study by Jangid et al. (2007), LuxIR homologs are universally present in the genus *Aeromonas*. The LuxR homologs in Aeromonas species showed a wide range of similarity, from 79.28 to 100%. In contrast, the LuxI homologs showed lower sequence similarity which ranged from 69.34 to 100%. From *in silico* analysis of strain 159 and closely-related species, all *Aeromonas* spp. are found to possess single copy of LuxI homologs (data not shown). In terms of AHL profile, C4-HSL is found to be the major AHL produced by both AhyI and AsaI. Apart from C4-HSL, minute amount of C6-HSL was identified from the spent supernatant of both *A. hydrophila* and *A. salmonicida* (Swift et al., 1997). In fact, the production of C4-HSL and C6-HSL by AhyI was detected to be in approximate ratio of 70:1. In contrast, C6-HSL is not found in the spent supernatant of *A. veronii* strain 159. Besides C4-HSL and C6-HSL, C5-HSL is another AHL significantly detected from many *A. hydrophila* strains from clinical samples (Chan et al., 2011). In this study, the production of only a single type of AHL, i.e. C4-HSL and the presence of a single copy of the QS genes in strain 159 highly suggests a tight regulation in pathogenesis by a singular type of autoinducer.

In conclusion, the whole genome sequencing of strain 159 enables the prediction of DNA sequences of target genes, i.e., QS-related genes. The findings of AHL-based QS system in strain 159 have placed further interest to explore its role in the mechanism of pathogenesis. This could possibly provide a model for bacterial cell-cell communication among *Aeromonas* species and hence a potential antimicrobial target in treating *Aeromonas* infections.

## Author contributions

XC and KH conceived and designed the experiments; XC performed the experiments and analyzed the data; KH and WY wrote the paper; KC edited and approved the manuscript.

### Conflict of interest statement

The authors declare that the research was conducted in the absence of any commercial or financial relationships that could be construed as a potential conflict of interest.
